# Vertical Transmission of *Babesia microti*, United States

**DOI:** 10.3201/eid1808.110988

**Published:** 2012-08

**Authors:** Julie T. Joseph, Kerry Purtill, Susan J. Wong, Jose Munoz, Allen Teal, Susan Madison-Antenucci, Harold W. Horowitz, Maria E. Aguero-Rosenfeld, Julie M. Moore, Carlos Abramowsky, Gary P. Wormser

**Affiliations:** New York Medical College, Valhalla, New York, USA (J.T. Joseph, K. Purtill, J. Munoz, H.W. Horowitz, M.E. Aguero-Rosenfeld, G.P. Wormser);; New York State Department of Health, Albany, New York, USA (S.J. Wong, A. Teal, S. Madison-Antenucci);; University of Georgia, Athens, Georgia, USA (J.M. Moore);; and Emory University School of Medicine, Atlanta, Georgia, USA (C. Abramowsky)

**Keywords:** Babesia microti, bacteria, transmission, babesiosis, infant, placenta, mother, newborn, New York, United States

## Abstract

Babesiosis is usually acquired from a tick bite or through a blood transfusion. We report a case of babesiosis in an infant for whom vertical transmission was suggested by evidence of *Babesia* spp. antibodies in the heel-stick blood sample and confirmed by detection of *Babesia* spp. DNA in placenta tissue.

Babesiosis is an emerging infection in the United States, principally caused by *Babesia microti* (*1*). The most common route of infection is the bite of an *Ixodes scapularis* tick; transmission can also occur by transfusion of infected blood products, and vertical transmission in animals has been documented (*2**,**3*) and is a potential route of transmission for humans. We report a case of babesiosis in an infant for whom vertical transmission was suggested by *Babesia* spp. antibodies in a heel spot blood sample and confirmed by detection of *Babesia* DNA in placenta tissue.

## The Case-Patient

A 6-week-old girl from Yorktown Heights, New York, was admitted to the hospital on September 16, 2002, with a 2-day history of fever, irritability, and decreased oral intake. The mother was asymptomatic during and after her pregnancy. The infant was delivered vaginally and full term at 3,430 g without complications. The infant’s mother had visited parks in Westchester and Dutchess Counties in New York during the pregnancy but was unaware of any tick bites. The infant had no known tick exposure, and neither mother nor infant had a history of blood transfusion.

During examination, the infant was alert but irritable and pale. Axillary temperature was initially 36.8°C but increased to 38.1°C on the same day. Her conjunctivae were icteric, she had a palpable spleen tip, and her liver was palpable 3 cm below the costal margin. Initial laboratory findings included hemoglobin 7.1 g/dL, platelet count 100 × 10^3^/μL, and leukocyte count 19.7 × 10^3^ cells/μL with a differential of 4% segmented neutrophils, 80% lymphocytes, and 16% monocytes. Reticulocyte count was 5.5%. Total bilirubin concentration was 2 mg/dL with a direct fraction of 0.4 mg/dL; aspartate aminotransferase level was 66 U/L, alanine aminotransferase level was 50 U/L, and alkaline phosphatase level was 339 U/L. Cultures of blood, urine, and cerebrospinal fluid samples yielded negative results. Lyme disease serologic test result was negative.

Routine examination of a peripheral blood smear showed *B. microti* in 4% of erythrocytes (Figure); a blood sample from the infant was positive by PCR for *B. microti* DNA. Total *B. microti* antibody titer was >256 by indirect immunofluorescence assay, with a polyvalent secondary antibody (anti-IgG+IgA+IgM) (*4*) that was presumed to be principally IgG because test results for IgM were negative (Technical Appendix). The heel-stick blood sample obtained on the infant’s third day of life as part of newborn screening was tested and found to be negative for *B. microti* by PCR (*5*) and for IgM but total antibody positive (>128) (Technical Appendix).

**Figure:**
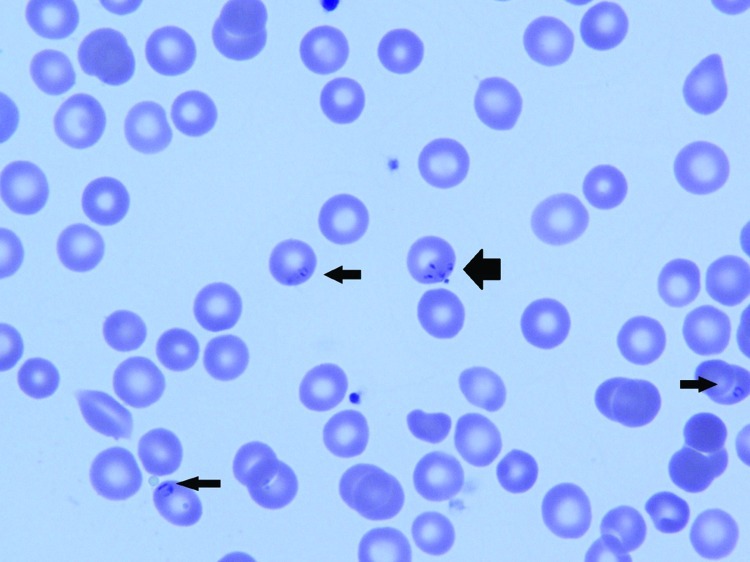
Peripheral blood smear of 6-week-old infant with suspected congenital babesiosis. Thin arrows indicate *Babesia* spp. parasites; thick arrow shows the classic tetrad formation or Maltese cross.

Examination of the placenta showed focal basal decidual inflammation, mild chorangiosis, and villus dysmaturity. *Babesia* spp. piroplasms were not detected in maternal or fetal blood by histologic examination of hematoxylin and eosin–stained sections of formalin-fixed, paraffin-embedded tissue of the placenta disk, amnion/chorion, and umbilical cord. *Babesia* DNA was detected by real-time PCR testing of paraffin-embedded placenta tissue (Technical Appendix) (*6*). Cycle threshold values were relatively high (37.1–38.2), indicating that the amount of parasite DNA in the sample was close to the limit of detection; results were reproducible on duplicate testing of DNA samples extracted from separate paraffin blocks. The real-time PCR product was of the correct size, and the melting curve demonstrated melting temperatures within 1°C from the placenta, the positive control, and a positive sample from an unrelated patient , confirming that the correct product was amplified. At time of the illness in the infant, the mother was negative for *Babesia* spp. according to PCR and smear but positive for total antibodies (>256).

The infant was treated with a 9-day course of azithromycin plus atovaquone. A blood transfusion was administered when her hemoglobin concentration fell to 5.2 g/dL. The infant became afebrile by 72 hours and was discharged after a 5-day hospitalization. Repeat blood smears revealed a parasite load of 0.3% at discharge. On final evaluation at 22 months of age, physical examination revealed no abnormalities; hemoglobin level was 11.7 g/dL, *Babesia* PCR was negative, and total *Babesia* antibody level was positive at 128.

## Conclusions

Congenital babesiosis has been rarely reported (Table) (*7**–**10*). This case provided convincing evidence for congenital babesiosis because of prepartum infection involving the placenta in the mother. On the basis of experience with congenital malaria, we assume that *Babesia* spp. parasites cross the placenta during pregnancy or at the time of delivery (*11**,**12*). In congenital malaria, increasing evidence suggests that the malaria parasites are most often acquired antenatally by transplacental transmission of infected erythrocytes (*12*).

**Table:** Comparison of selected clinical and laboratory data from reported cases of congenital babesiosis in 5 infants*

Clinical data	Reference				
	(*7*)	(*8*)	(*9*)	(*10*)	This study
Year of diagnosis/ location	Not given/Long Island, New York	Not given/Long Island, New York	Not given/New Jersey	Not given/Long Island, New York	2002/Westchester County, New York
Infant age at time of symptom onset, d	30	32	19	27	41
Clinical findings	Fever, irritability, pallor, hepatosplenomegaly	Fever, lethargy, poor feeding, pallor, scleral icterus, hepatomegaly	Fever, poor feeding, gagging, irritability, pallor, scleral icterus, hepato-splenomegaly	Fever, pallor	Fever, decreased oral intake, irritability, scleral icterus, pallor, hepatosplenomegaly
Initial babesia parasitemia level, %	5	4.4	15	2	4
Hospitalization, d	6	5	8	NA	5
Maternal tick bite	1 wk before delivery	7 wk before delivery	4 wk before delivery	None known	None known
*Babesia* spp. serologic and PCR results for infant	30 d after birth: IgM+/IgG+ (128/128) by IFA; 32 d after birth: IgM+/IgG+ (256/512) by IFA; PCR ND	At illness onset: IgG IFA 160; IgM/IgG immunoblot +; PCR ND	At illness onset: IgM+/IgG+ (40/256) by IFA; PCR ND	NA	Newborn screening (heel stick): IgM– (<16); total antibody + (>128) by IFA; PCR–; 6 wks after birth: IgM– (<16); total antibody + (>256) by IFA; PCR+
*Babesia* spp. evaluation results for mother	30 d after birth: IgM+/IgG+ (2,048/1,024); 32 d after birth: IgM+/ IgG+ (4,096/1,024); peripheral smear – at time of delivery and at 30 and 32 d after birth	7 wk before birth: IgG IFA <40; IgM/IgG immunoblot –; 2 mo after birth: IgG IFA 640; IgM/IgG immunoblot +; peripheral smear – at delivery and at infant illness onset	At infant illness onset: IgM+/IgG+ (80/>1,024) by IFA; peripheral smear negative at time of infant illness onset	At infant illness onset: PCR+	Birth: placenta PCR+; 6 wk after birth: IgM ND; total antibody + (>256) by IFA; PCR–; peripheral smear –
HGB, g/dL	9.3	10.8	8.8	NA; HCT 24.3%	7.1
Platelets, x 10^3^/μL	38	87	34	101	100
Leukocytes/PMN leukocytes, cells/μL	6,500/1,170	NA	9,000/1,890	NA	19,700/788
LDH, U/L	894	NA	2535	NA	NA
Bilirubin indirect, mg/dL	3.6	9.7	5.9	NA	1.6
AST, U/L	90	NA	53	NA	66
ALT, U/L	90	NA	18	NA	50
Treatment	CLI and quinine for 10 d	CLI and quinine with AZT added on day 3; on day 5 changed to AZT plus quinine for additional 7 d	AZT and ATO for 10 d	AZT and ATO, duration not given	AZT and ATO for 9 d
Follow-up	Well at 6 mo posttreatment	Improved at 2 wk	Lost to follow-up	NA	22 mo
Blood transfusion for anemia	Yes, for HCT of 18%	Yes, for HGB of 7.3 g/dL	Yes, for HGB of 7.0 g/dL	Yes, for HCT of 17.3%	Yes, for HGB of 5.2 g/dL with HCT of 15.8%

*No mothers became ill. PMN, polymorphonuclear; AST, aspartate aminotransferase; ALT, alanine aminotransferase; LDH, lactate dehydrogenase level; HCT, hematocrit; HGB, hemoglobin; IFA, indirect immunofluorescence assay. CLI, clindamycin; AZT, azithromycin; ATO, atovaquone. NA, not available; ND, not done. +, positive; –, negative.

Reported cases of congenital babesiosis share many similarities, including asymptomatic maternal infection and development of fever, hemolytic anemia, and thrombocytopenia in the infant detected between 19 and 41 days after birth. All of the infants responded to antimicrobial drug therapy; 3 were treated with azithromycin plus atovaquone (*9**,**10*), the preferred treatment regimen for mild babesiosis (*1*). All infants required a blood transfusion because of severe anemia. The clinical signs and symptoms for these cases of congenital babesiosis are similar to those of congenital malaria in non–disease endemic areas (*11**,**13*).

We found *Babesia* spp. antibodies on day 3 of life by analyzing the patient’s heel-stick blood sample, which likely represented maternal transfer of IgG. Passive transfer of maternal antibodies is regarded as a protective factor against congenital malaria, and some newborns with malaria who are parasitemic at birth spontaneously clear the infection without ever becoming ill (*11**,**14*). The temporary presence of maternal IgG in infants has been suggested as an explanation for the typical 3–6 week incubation period of congenital malaria in non–disease endemic areas (*14*).

The real-time PCR used to find *B. microti* DNA in placenta tissue is ≈20× more sensitive than microscopic examination of Giemsa-stained blood smears (*6*). Assuming a blood sample with a parasitemia equivalent to that detected in the placental tissue, a blood smear would contain <10 infected cells per slide. Given the low level of *Babesia* DNA in the placenta tissue, it is not surprising that histologic examination did not reveal piroplasms. Nonetheless, limited evidence of placental abnormalities suggests a pathologic process.

In summary, babesiosis is an emerging infectious disease (*15*) that can rarely cause congenital infection. This diagnosis should be considered in the differential diagnosis of fever and hemolytic anemia in infants from disease-endemic areas.

Technical Appendix Detailed methods of testing for *Babesia* spp. in 6-week-old infant with suspected congenital babesiosis.
