# Supplementary material for: Vertical Transmission of Babesia microti, United States
Source: Emerg Infect Dis. 2012 Aug;18(8):1318–21. doi: 10.3201/eid1808.110988 (PMC3414010; doi:10.3201/eid1808.110988)
Supplement: Technical Appendix — Detailed methods of testing for Babesia spp. in 6-week-old infant with suspected congenital babesiosis. [file 11-0988_Techapp-s1.pdf]

Article DOI: http://dx.doi.org/10.3201/eid1808.110988

# Vertical Transmission of *Babesia microti*, United States

## Technical Appendix

### Antibody testing of the infant's blood spot

Approximately one-fourth of the blood spot (three 1/8 inch diameter punches) was eluted in 140 µL of 10 g/L goat serum (Sigma) in PBS overnight at 4°C. The eluate was filtered through a 0.45-µm syringe filter and then tested in a polyvalent (IgM, IgA, IgG) indirect immunofluorescence assay (IFA) for *Babesia* spp. antibodies. Controls were made by spotting both *Babesia* antibody negative whole blood and the same blood spiked with a positive control serum. The spots were allowed to dry overnight and eluted as above. The IFA used *Babesia* spp.–infected golden hamster erythrocytes fixed to glass slides. The conjugate was polyvalent FITC-conjugated anti-human antibody (Kirkegaard & Perry Laboratories, Gaithersburg, MD) in 0.1% Evans blue dye (*1*). For detection of IgM antibody specifically, the eluates were treated with Gullsorb (Meridian Diagnostics, now Meridian Bioscience, Cincinnati, OH) to remove IgG and then retested using the same IFA procedure with an IgM-specific FITC-conjugate (KPL). The controls achieved the expected results.

### DNA extraction and conventional PCR

DNA was extracted from EDTA-preserved whole blood, 3 mm diameter punches of the dried blood spot, and from freshly cut 10-µm sections of paraffin-embedded tissue, using protocols for blood, dried blood spots, and FFPE Tissue respectively (QIAGEN). Conventional PCR was performed on the extracts from the blood spot and whole blood specimen essentially as previously described targeting the 18S rDNA of *B. microti* (*2*), except the primer sets were used independently to produce two amplification products. Amplification of the human β-globin gene was performed to test for inhibition. The positive control was a plasmid containing the rDNA target, spiked into a negative blood sample and extracted in parallel with the patient specimen.

Cycling conditions were: 95°C 10 min, 35 cycles (94°C 1 min, 58°C 1 min, 72°C 1 min), with a final extension at 72°C for 4 min.

## Real-Time PCR

DNA extracted from the paraffin embedded tissue was assayed for the presence of *B. microti* using a recently developed real-time PCR assay targeting the 18S rRNA gene (*6*). Extracted DNA (15 µl) was amplified using FastStart DNA Master mix without ROX (Roche) in a 25 µl reaction mixture with 5.5 mM MgCl2, 500 nM primers (forward-CAGGGAGGTAGTGACAAGAAATAACA and reverse-GGTTTAGATTCCCATCATTCCAAT, and 250 nM probe (6FAM-TACAGGGCTTAAAGTCT-MGBNFQ). A 2-step PCR cycling protocol (95°C 10 min, and 45 cycles of 95°C 15 s, 60°C 1 min) was used to amplify a 72-bp product. An internal control was used to evaluate the efficiency of extraction and test for inhibition of the sample, as described elsewhere (*3*).

### References


1. Chisholm ES, Ruebush TK II, Sulzer AJ, Healy GR. *Babesia microti* infection in man: evaluation of an indirect immunofluorescent antibody test. Am J Trop Med Hyg. 1978;27:14–9. PubMed

2. Persing DH, Mathiesen D, Marshall WF, Telford SR, Spielman A, Thomfod JW, et al. Detection of *Babesia microti* by polymerase chain reaction. J Clin Microbiol. 1992;30:2097–103. PubMed

3. Teal AE, Habura A, Ennis J, Keithly J, Madison-Antenucci S. A new real-time PCR assay for improved detection of the parasite *Babesia microti*. J Clin Microbiol. 2012;50:903–8. PubMed http://dx.doi.org/10.1128/JCM.05848-11
